# *Leishmania* Promastigotes Enhance Neutrophil Recruitment through the Production of CXCL8 by Endothelial Cells

**DOI:** 10.3390/pathogens10111380

**Published:** 2021-10-26

**Authors:** Sarah D’Alessandro, Silvia Parapini, Yolanda Corbett, Roberta Frigerio, Serena Delbue, Annalisa Modenese, Marina Gramiccia, Pasquale Ferrante, Donatella Taramelli, Nicoletta Basilico

**Affiliations:** 1 Dipartimento di Scienze Farmacologiche e Biomolecolari, Università degli Studi di Milano, Via Pascal, 36, 20133 Milano, Italy; sarah.dalessandro@unimi.it (S.D.); ycorbett179@gmail.com (Y.C.); donatella.taramelli@unimi.it (D.T.); 2 Dipartimento di Scienze Biomediche per la Salute, Università degli Studi di Milano, Via Pascal, 36, 20133 Milano, Italy; silvia.parapini@unimi.it; 3 Dipartimento di Scienze Biomediche, Chirurgiche e Odontoiatriche, Università degli Studi di Milano, Via Pascal, 36, 20133 Milano, Italy; roberta.frigerio@unimi.it (R.F.); serena.delbue@unimi.it (S.D.); pasquale.ferrante@ic-cittastudi.it (P.F.); 4 Laboratorio Analisi, Istituto Clinico Città Studi, 20131 Milano, Italy; annalisa.modenese@ic-cittastudi.it; 5 Department of Infectious Diseases, Unit of Vector-Borne Diseases, Istituto Superiore di Sanità, Viale Regina Elena 299, 00161 Rome, Italy; marina.gramiccia@iss.it

**Keywords:** *Leishmania* promastigotes, endothelial cells, CXCL8, neutrophils

## Abstract

Endothelial cells represent one of the first cell types encountered by *Leishmania* promastigotes when inoculated into the skin of the human hosts by the bite of phlebotomine sand flies. However, little is known on their role in the early recruitment of phagocytic cells and in the establishment of the infection. Initially, neutrophils, rapidly recruited to the site of promastigotes deposition, phagocytize *Leishmania* promastigotes, which elude the killing mechanisms of the host cells, survive, and infect other phagocytic cells. Here, we show that *Leishmania* promastigotes co-incubated with HMEC-1, a microvascular endothelial cell line, exhibited significant morphological changes and loss of infectivity. Moreover, promastigotes of different *Leishmania* species stimulated the production of CXCL8 by HMEC-1 in a dose- and TLR4-dependent manner. Interestingly, we observed that the conditioned media from *Leishmania*-stimulated HMEC-1 cells attracted leukocytes, mostly neutrophils, after 2 h of incubation. After 24 h, a higher percentage of monocytes was detected in conditioned media of unstimulated HMEC-1 cells, whereas neutrophils still predominated in conditioned medium from *Leishmania*-stimulated cells. The same supernatants did not contain CCL5, a chemokine recruiting T cells and monocytes. On the contrary, inhibition of the production of CCL5 induced by TNF-α was seen. These data indicate that the interaction of *Leishmania* promastigotes with endothelial cells leads to the production of chemokines and the recruitment of neutrophils, which contribute to the establishment of *Leishmania* infection.

## 1. Introduction

Leishmaniasis are parasitic neglected vector-borne diseases, caused by protozoa of the genus *Leishmania*. Worldwide, an estimated 700,000 to 1 million new cases occur every year [1]. These diseases are transmitted to the vertebrate host by the bite of an infected female sand fly. Human infections are caused by more than 20 of the 30 species of *Leishmania* that infect mammals. During blood feeding, *Leishmania* metacyclic flagellated promastigotes are co-inoculated into the host’s skin together with sand fly saliva and midgut content. Sand flies damage the skin of the vertebrate hosts using their proboscis to rip and scratch through the tissues causing bleeding, which immediately activates host hemostatic and inflammatory mechanisms. Sand fly saliva contains components with anticoagulant and anti-inflammatory properties, which counteract the host’s defense systems. Promastigotes are then ingested by phagocytic cells, where they develop into obligate, non-motile amastigotes [2].

Several reports indicate that neutrophils are the first leukocyte population recruited to and infected at the site of *Leishmania* deposition [3,4,5,6]. This type of exudate was first observed by Wilson et al. [7] 1 h after intradermal inoculation of *L. donovani* promastigotes in the hamster model. Later, Pompey et al. [8] and Beil et al. [9] confirmed in experimental murine infections that a neutrophil infiltrate is present 3 h after subcutaneous inoculation of *L. amazonensis* or *L. major* promastigotes. Chemokines attracting first neutrophils and subsequently NK cells to the site of *L. major* infection in mice were also described by Muller et al. [10]. The neutrophils’ chemotactic stimuli seem to derive from the surrounding tissue and from the sand fly saliva [11,12]. Moreover, it has also been reported that gut microbes from the sand fly are egested alongside *Leishmania* parasites, triggering the inflammasome and the production of interleukin-1β, which sustains neutrophil infiltration [13].

Being the most abundant leukocytes in blood, neutrophils play an important role in innate immunity and in the modulation of adaptive immunity [14]. Although they possess potent antimicrobial activity, they seem to play an unfavorable role in the development of leishmaniasis. Different studies have indicated that neutropenic mice have a better disease outcome than immunocompetent mice, suggesting a harmful role for neutrophils [3,9,10]. It has been reported that, once phagocytized, the parasites can elude the killing mechanisms of neutrophils and proliferate while being protected from other immune cells [15]. This seems to be favored by the prolonged survival of *Leishmania*-infected neutrophils and the delayed apoptosis demonstrated in vitro [16]. Parasites use neutrophils to be finally ingested by macrophages, their final host, where they replicate and infect new phagocytic cells [17].

Chemokines are chemotactic cytokines involved in the recruitment of leukocytes through binding of chemokine receptors, which activate different biological functions such as integrin activation and cell migration [18]. Members of the CXC chemokines, such as CXCL8 (also known as IL-8), act mainly on neutrophils, whereas members of the CC chemokines, such as CCL5 (also known as RANTES), attract different cells, including monocytes, lymphocytes, basophils and eosinophils, but not neutrophils [19]. Depending on the chemokines produced, the inflammatory infiltrate may vary, as well as its role in the control or progression of the infection. Although endothelial cells represent one of the first cell types encountered by *Leishmania* promastigotes after their deposition in the human skin, their role in the establishment of the infection is still unclear. Since endothelial cells are an important source of inflammatory mediators, including chemokines, we investigated whether endothelial cells may play a role in the neutrophil recruitment in the presence of *Leishmania* promastigotes.

## 2. Results

### 2.1. HMEC-1 Viability in the Presence of Leishmania spp.

**Figure 1:**
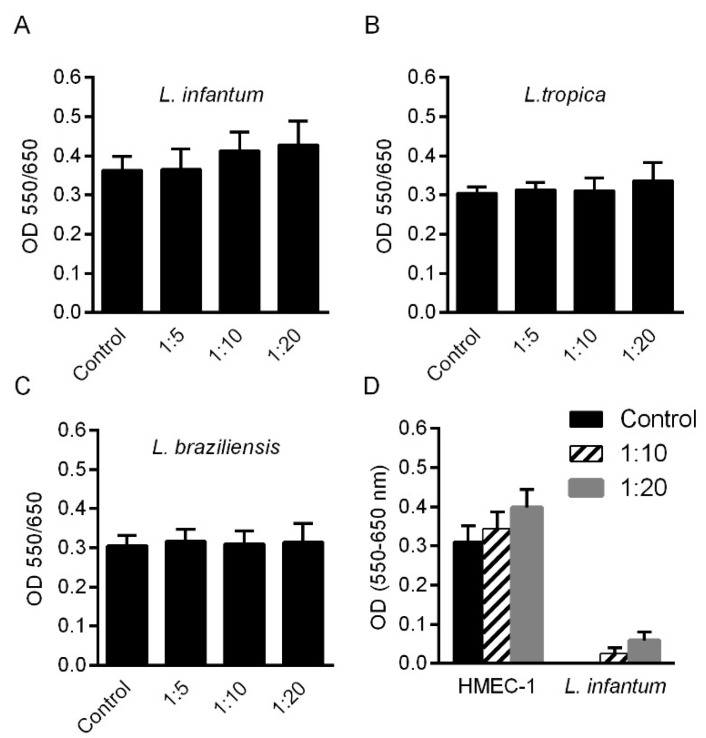
HMEC-1 viability after 24 h of co-incubation with *Leishmania* spp. Cells were left untreated (control) or treated for 24 h with promastigotes of *L. infantum* (**A**), *L. tropica* (**B**) or *L. braziliensis* (**C**) at different cell:promastigote ratios (1:5, 1:10, 1:20). Promastigotes of *L. infantum* were cocultured with HMEC-1 or were grown alone for 24 h (**D**). Cell viability was measured by MTT assay. Data are expressed as OD (550/650) means ± standard deviation (SD) of three independent experiments. One-way ANOVA, Tukey’s multiple comparisons.

Endothelial cells were co-cultured with promastigotes of *L. infantum*, *L. tropica* or *L. braziliensis* at different cell: *Leishmania* ratios for 24 h and HMEC-1 viability was measured by MTT assay. As shown in Figure 1, cell viability was unaffected by the presence of promastigotes. The slight, insignificant, increase in MTT reduction, occurring in the presence of high concentration of *Leishmania* promastigotes was due to the ability of the parasites themselves to metabolize MTT, as shown in Figure 1D, where *L. infantum* promastigotes alone or in the presence of HMEC-1 were incubated for 24 h and viability was evaluated by MTT assay. Approximately, 10–20% of MTT values can be attributed to *Leishmania* promastigotes.

### 2.2. Viability, Morphological Analysis and Infectivity of Leishmania spp. in HMEC-1 Culture Conditions

**Figure 2:**
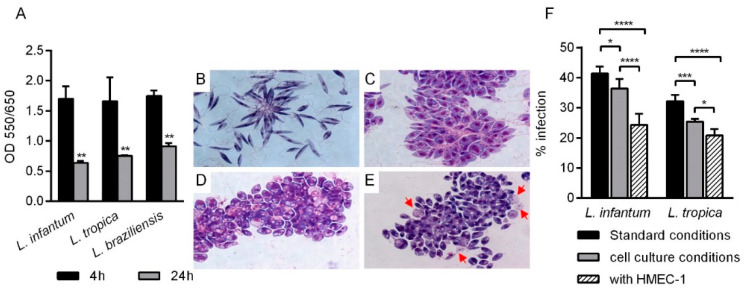
*Leishmania* spp. viability, morphology and infectivity after incubation in different culture conditions. (**A**) Promastigotes of *L. infantum*, *L. tropica* or *L. braziliensis* (5 × 10^6^ parasite/mL) were distributed in 96-well round bottom microplates (100 μL/well) and incubated for 4 or 24 h in cell culture conditions (cell medium, 37 °C, 5% CO_2_). *Leishmania* viability was measured by the MTT assay. Data are from a representative experiment and are expressed as OD (550/650) means ± standard deviation (SD) of four replicates. ** *p* > 0.01 24 h vs. 4 h 2-way ANOVA, Sidak’s multiple comparisons test. (**B**–**E**) For the morphological analysis, promastigotes of *L. infantum* were seeded in 24-well flat bottom tissue culture clusters at 10^6^ parasites/well and cultured in different conditions. After incubation, Giemsa smears were prepared and analyzed under light microscopy. Promastigotes of *L. infantum* cultured in cell culture conditions (in the absence of HMEC-1 cells) for 0 (**B**), 4 (**C**) or 24 (**D**) h; (**E**) promastigotes of *L. infantum* co-cultured with HMEC-1 in cell culture conditions (cell medium, 37 °C, 5% CO_2_) for 24 h. Red arrows indicate parasites with pale cytoplasm and broken cell membranes. (**F**) PMA-differentiated THP-1 cells plated in 16-chamber Lab-Tek culture slides were infected with *L. infantum* or *L. tropica* promastigotes (1:10 cell:promastigote ratio) for 24 h. Promastigotes were previously incubated in: (i) standard conditions (complete Schneider’s Drosophila Medium, 23 °C); (ii) cell culture conditions (cell medium, 37 °C, 5% CO_2_); (iii) co-incubation with HMEC-1 (1:10, cell:promastigote ratio) under cell culture conditions. At the end of incubation, cells were fixed, stained with Giemsa and the percentage of infected macrophages was determined by counting infected cells under light microscopy. Data are from two experiments performed in quintuplicate. * *p* > 0.05; *** *p* > 0.001; **** *p* > 0.0001 1-way ANOVA, Dunnett’s multiple comparisons test.

In the first set of experiments, promastigotes from different *Leishmania* spp. were incubated under endothelial cell culture conditions (HMEC-1 culture medium, 37 °C and 5% CO_2_) for 4 or 24 h and their viability was determined by MTT assay and light-microscopy examination. The optical density was significantly lower at 24 h than at 4 h of incubation (*p* > 0.01), indicating a reduced viability or a reduced proliferation of promastigotes in the conditions used for in vitro HMEC-1 culturing (Figure 2A). Similar effects were observed for all three different species examined. MTT data were further confirmed by microscopic parasite counting. Starting from a concentration of 5 × 10^6^ *Leishmania*/mL, after 4 h of incubation in cell culture conditions, parasites reached 15 × 10^6^/mL and 14 × 10^6^/mL at 23 °C and 37 °C, respectively. After 24 h of incubation, parasites were 32 × 10^6^/mL and 12 × 10^6^/mL at 23 °C and 37 °C, respectively. These data confirm that promastigotes do not proliferate at 37 °C, and some of them die.

*L. infantum* morphology was evaluated by Giemsa smears at different incubation times (0, 4, 24 h) in cell culture conditions (Figure 2B–D). After 4 h, some *Leishmania* promastigotes appeared enlarged with a visible flagellum, whereas some others were smaller, rounded forms with a short flagellum (Figure 2C). After 24 h, all the parasites appeared rounded with short or even with no visible flagellum (Figure 2D). The same morphology was observed when *Leishmania* promastigotes were co-incubated with HMEC-1 (Figure 2E). In addition, some parasites appeared with a pale cytoplasm and broken cell membranes (red arrows in Figure 2E).

Next, in order to assess whether the selected incubation conditions might also alter promastigotes infectivity, the ability of *L. infantum* or *L. tropica* promastigotes (1:10, cell:parasite ratio) to infect PMA differentiated THP-1 macrophages was examined after 4 h of incubation in different culture conditions: (i) standard parasite conditions (complete Schneider’s Drosophila Medium, 23 °C); (ii) cell culture conditions (HMEC-1 medium, 37 °C and 5% CO_2_); (iii) co-incubation with HMEC-1 in cell culture conditions. The percent of infected macrophages was then evaluated after 24 h of incubation. Figure 2F clearly shows that cell culture conditions and, especially, co-incubation with HMEC-1, significantly impaired the parasites’ ability to infect human macrophages.

### 2.3. CXCL8 Production by Endothelial Cells Treated with Leishmania spp. Promastigotes

**Figure 3:**
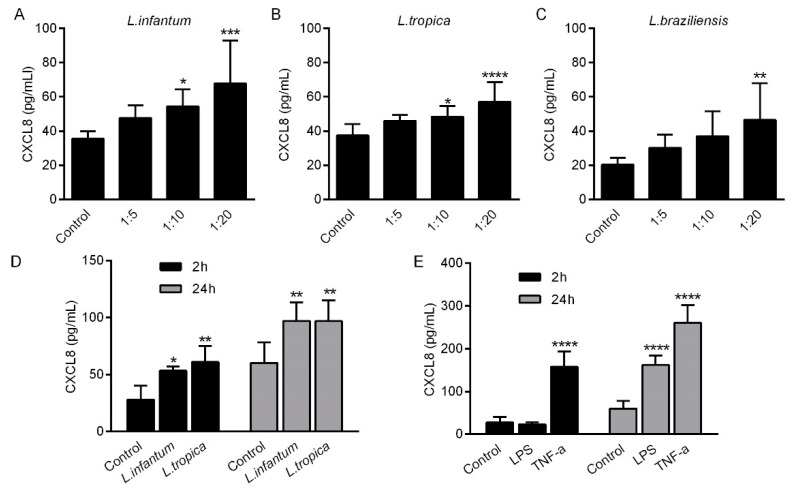
HMEC-1 were left untreated (control) or co-incubated with promastigotes of *L. infantum*, *L. tropica* or *L. braziliensis* at different cell: *Leishmania* ratios for 24 h. Thereafter, CXCL8 levels were measured in cell supernatants. Exposure of HMEC-1 to promastigotes of *Leishmania* spp. stimulated the production of CXCL8 in a concentration-dependent manner (**A**–**C**). A cell: *Leishmania* ratio of 1:10 was sufficient for inducing significant amount of CXCL8, compared to untreated control cells. Time course experiments indicated that after 2 h of co-incubation, a significant amount of CXCL8 was induced by both *L. infantum* and *L. tropica* (**D**). When LPS or TNF-α were used as positive controls, only TNF-α, but not LPS, induced significant levels of CXCL8 after 2 h of stimulation (**E**). Data are expressed as the mean ± standard deviation of three independent experiments. * *p* > 0.05; ** *p* > 0.01; *** *p* > 0.001; **** *p* > 0.0001 vs. control 2-way ANOVA, Tukey’s multiple comparisons test.

HMEC-1 were left untreated (control) or co-incubated with promastigotes of *L. infantum*, *L. tropica* or *L. braziliensis* at different cell: *Leishmania* ratio for 24 h. Thereafter, CXCL8 levels were measured in cell supernatants. Exposure of HMEC-1 to promastigotes of *Leishmania* spp. stimulated the production of CXCL8 in a concentration-dependent manner (Figure 3A–C). A cell: *Leishmania* ratio of 1:10 was sufficient for inducing significant amount of CXCL8, compared to untreated controls. In addition, time course experiments indicated that after 2 h of co-incubation, a significant amount of CXCL8 was induced by both *L. infantum* and *L. tropica* (Figure 3D). When, LPS or TNFα, were used as positive controls, only TNFα, but not LPS, induced significant levels of CXCL8 after 2 h of stimulation (Figure 3E).

**Figure 4:**
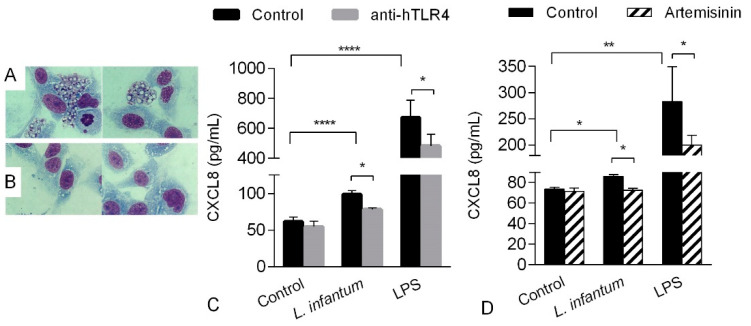
HMEC-1 were co-incubated with *L. infantum* for 24 h in chamber slides. Cell were fixed with methanol and stained with Giemsa immediately (**A**) or extensively washed with PBS to remove free *Leishmania* parasites (**B**). (**C**) CXCL8 production by HMEC-1 incubated with promastigotes of *L. infantum* in the presence of an anti-TLR4 antibody. Cells were left untreated (control) or incubated with or without 0.5 µM of an anti-TLR4 antibody (hTLR4) before the addition of *L. infantum* promastigotes or LPS for 24 h. Data are expressed as the mean ± standard deviation (SD) of three independent experiments. (**D**) CXCL8 production by HMEC-1 incubated with promastigotes of *L. infantum* in the presence of artemisinin 10 µM. Data show one representative experiment. CXCL8 levels released in cell supernatants were measured by ELISA. * *p* > 0.05; ** *p* > 0.01; **** *p* > 0.0001 vs. control 2-way ANOVA, Tukey’s multiple comparisons test.

To verify whether CXCL8 production was induced by phagocytosis of *Leishmania* promastigotes, HMEC-1 were co-incubated with *L. infantum* for 24 h. Cell monolayers were then extensively washed to remove non-internalized parasites and further stained with Giemsa. As shown in Figure 4, before washing, many *Leishmania* promastigotes were present around endothelial cells (Figure 4A). However, after washing, all *Leishmania* promastigotes were successfully removed, showing that cells were not infected by parasites (Figure 4B).

Since *Leishmania* promastigotes can interact with Toll-like receptors (TLRs), the involvement of TLR4 in the production of CXCL8 from *Leishmania*-induced HMEC-1 was subsequently evaluated. In the presence of an anti-TLR4 antibody, the production of CXCL8 induced by *Leishmania infantum* or LPS (the main ligand of TLR4) was reduced by 20.4 and 28.3%, respectively (Figure 4C). Since TLR- 4 activates the NF-κB pathway to regulate the expression of proinflammatory mediators, artemisinin, a known inhibitor of NF-κB was used. Artemisinin reduced the *Leishmania*-induced CXCL-8 production by 16.8%. When LPS was used as positive control, artemisinin induced a significant reduction of CXCL-8.

**Figure 5:**
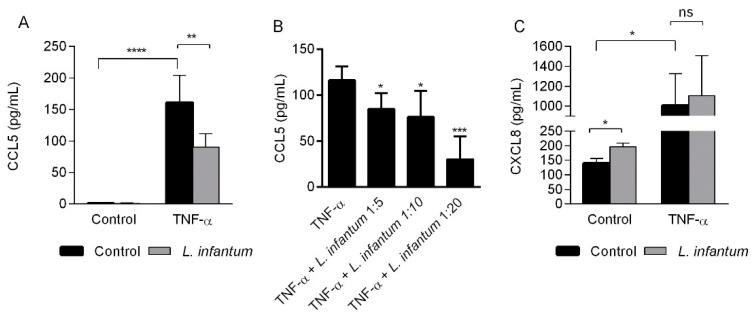
CCL5 and CXCL8 production by HMEC-1 incubated with promastigotes of *L.* *infantum*. Cells were left untreated (control) or incubated for 24 h with *L. infantum* promastigotes (1:10, cell:parasite ratio) in the presence or absence of TNF-α (100 U/mL) (**A**). Cells were incubated for 24 h with promastigotes of *L. infantum* at different cell:promastigote ratios (1:5, 1:10, 1:20) in the presence of TNF-α (**B**). CCL5 levels in cell supernatants were measured by ELISA. Data are the mean ± standard deviation (SD) of three independent experiments. * *p* < 0.05; ** *p* < 0.01; *** *p* < 0.001; **** *p* < 0.0001 vs. control, 2-way ANOVA, Sidak’s (**A**) or Tukey’s (**B**) multiple comparisons test. (**C**) Cells were left untreated (control) or incubated for 24 h with promastigotes of *L. infantum* (1:10, cell:parasite ratio) in the presence or absence of TNF-α (100 U/mL) (**C**). CXCL8 levels were measured by ELISA in cell supernatants. Data are the mean ± standard deviation (SD) of three independent experiments. * *p* < 0.05; ns = not significant vs. control, 2-way ANOVA, Sidak’s multiple comparisons test.

Unlike CXCL8, promastigotes of *L. infantum* did not alter the basal production of CCL5 (Figure 5A), although they reduced the production of CCL5 induced by TNF-α in a dose-dependent manner (Figure 5B). This effect seems specific for CCL5 since *L. infantum* did not reduce the production of CXCL8 induced by TNF-α (Figure 5C).

### 2.4. Neutrophils Recruitment by Supernatant of Endothelial Cells Treated with Leishmania Promastigotes

**Figure 6:**
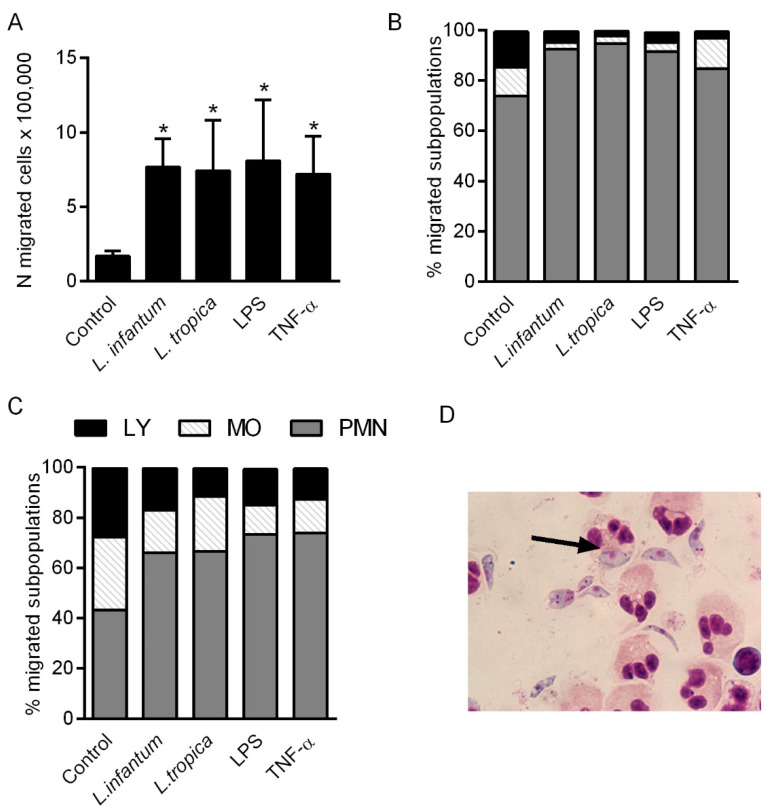
Percentage of leukocytes (**A**) and percentage of leukocyte subpopulations (**B**,**C**) migrated towards conditioned media from unstimulated or *Leishmania*-stimulated HMEC-1. Supernatants of cells stimulated with *L. infantum* or *L. tropica* promastigotes (1:10, cell:parasite ratio), LPS (100 ng/mL) or TNF-α (100 U/mL) for 24 h were collected and inserted into a transwell system. Human PBLs were then plated on top of the filter membrane and the migrated leukocytes were collected, counted and immobilized onto glass microscope slides after 2 h (**A**,**B**) and 24 h (**C**) of incubation at 37 °C. Slides were stained with Giemsa and the percentages of the different subpopulations were counted by light microscopy. * *p* > 0.05 vs. control, 1-way ANOVA, Dunnett’s multiple comparisons test. Neutrophils were recovered and incubated with *L. infantum* promastigotes. After incubation, both cells and parasites were recovered and Giemsa-stained smears prepared. Black arrow shows parasites inside neutrophils (**D**).

To verify whether the supernatants of the endothelial cells incubated with *Leishmania* promastigotes contain either active CXCL8, other chemokines or both, able to attract leukocytes, cell migration was evaluated in a transwell system using human PBLs and conditioned medium from HMEC-1 treated with *L. tropica* or *L. infantum* promastigotes. Conditioned medium from HMEC-1 alone or HMEC-1 stimulated with LPS or TNF-α were used as negative and positive controls, respectively. After 2 h of incubation, the number of migrated leukocytes and the percentage of the different leukocyte subpopulations was further evaluated. As shown in Figure 6A, the number of migrated leukocytes towards *Leishmania*-stimulated HMEC-1 conditioned medium was higher than those migrated to the conditioned medium from unstimulated control HMEC-1. The fold change in the mean of migrated cells towards *Leishmania* conditioned medium relative to unstimulated control cells ranged from 3.6 to 5.2 (*n* = 4) and 1.9 to 5.6 (*n* = 4) for *L. infantum* and *L. tropica*, respectively. The fold change for LPS and TNF-α relative to unstimulated cells ranged from 3.0 to 6.7 (*n* = 3) and from 2.9 to 5 (*n* = 3). The number of migrated leukocytes towards *Leishmania*-stimulated HMEC-1 conditioned medium was comparable to that induced by conditioned medium from TNF-α or LPS-stimulated HMEC-1, suggesting a strong production of functional chemokines induced by the parasites.

The percentages of the different migrated leukocyte subpopulations were then determined in each group. As shown in Figure 6B, after 2 h of migration, migrated cells were mostly neutrophils in all groups: 73% in medium from unstimulated cells, and more than 93% in *L. infantum* and *L. tropica* conditioned medium. After 24 h of chemotaxis, a higher percentage of monocytes and lymphocytes was observed in all groups. However, neutrophils continued to be the predominant subpopulation in conditioned media from stimulated cells (Figure 6C).

Neutrophils migrated after 2 h of chemotaxis were then recovered and incubated with *L. infantum* and *L. tropica* promastigotes in order to verify their ability to phagocytize parasites. After 30 min of incubation, some amastigotes were visible inside neutrophils (arrow in Figure 6D).

## 3. Discussion

After infected sand fly bites, neutrophils are rapidly recruited to the site of inoculation, representing the first cells infected by *Leishmania* promastigotes [10]. Still, the immune mechanisms governing the sustained and intensified neutrophil recruitment remain mostly undefined. It is expected that different factors deriving from the vector, parasite itself and host’s cells are all involved in the onset of infection. It is known that sandfly-derived factors, including salivary proteins and gut microbiota, act as chemoattractants for neutrophils [13,20]. Recently, it was demonstrated that members of sand fly yellow salivary proteins can induce in vitro chemotaxis of neutrophils [11]. Moreover, bacteria egested from sandflies activate the inflammasome along with the production of IL-1β, which acts as a chemotactic factor [13]. In the present study, we provide in vitro evidence that endothelial cells actively induce neutrophil chemotaxis by producing CXCL8, one of the most effective chemoattractants for neutrophils.

Metacyclic flagellated promastigotes, present in the anterior part of the midgut of the sand fly vector, differentiate into amastigotes inside the mammalian host’s phagocytic cells. This differentiation is modulated by environmental changes, such as pH and temperature, but also by H_2_O_2_ and iron uptake [21,22,23]. Indeed, changes in pH and temperature can induce loss of *Leishmania* viability through the production of reactive oxygen species [24]. Here, we also show in vitro, in cell culture conditions (mammalian cell culture medium, 37 °C, 5% CO_2_), that the parasites assume an amastigote-like morphology, stop to proliferate and some of them die. However, only 4 h in cell culture conditions resulted in a significant reduction in the parasites’ ability to infect macrophages. Infectivity was further decreased by co-culturing the parasites in the presence of HMEC-1. Therefore, even if the parasites assumed an amastigote-like morphology, they lost infectivity, suggesting that the differentiation process was not complete. This is consistent with the observation that amastigotes exhibit higher infectivity than promastigotes from the same *Leishmania* species [25]. The causes of this loss of infectivity albeit associated with an amastigote-like phenotype, are presently unknown. In co-culture conditions, endothelial cells may deprive the medium of the nutrients necessary for parasite viability and differentiation. Alternatively, HMEC-1 could produce toxic mediators affecting parasite viability. Most likely, however, the parasites’ loss of infectivity might reflect the fact that the parasites must quickly infect host cells and thus interact with cells present in the microenvironment, such as endothelial cells, to recruit phagocytic cells. We have indeed demonstrated that endothelial cells incubated with promastigotes of different *Leishmania* species produced CXCL8, a potent neutrophil chemotactic cytokine capable of delaying their apoptosis [16,26]. The induction of CXCL8 by *Leishmania* has also been demonstrated in a murine model and in human infections. Upon experimental infection with *L. major*, macrophage inflammatory protein (MIP)-2 and keratinocyte-derived cytokine (KC; also known as CXCL1), the functional murine homologues of human CXCL8 are rapidly produced in the skin [10]. Moreover, immunohistochemistry studies demonstrated strong expression of CXCL8 in dermal lesions of patients infected with *L. tropica* [27]. CXCL8 has also been shown to be produced by human monocytes stimulated by sandfly salivary gland homogenates [28]. Here, we showed that as little as 2 h of incubation in the presence of *Leishmania* promastigotes is enough for inducing CXCL8 secretion by HMEC-1. CXCL8 in endothelial cells is primarily stored in the secretory organelles, the Weibel–Palade bodies that can be rapidly exocytosed in response to different stimuli, such as thrombin and histamine [29]. The rapid release of CXCL8 from HMEC-1 may indicate that *Leishmania* promastigotes stimulate HMEC-1 to release the preformed chemokine present in Weibel–Palade organelles. This is different from LPS-induced production of CXCL8, which peaks after 24 h of incubation indicating neo-synthesis of the chemokine.

To investigate the mechanisms by which *Leishmania* promastigotes induce the production of CXCL8 by HMEC-1, the role of phagocytosis and of TLR4 receptor was investigated. Although endothelial cells are non-professional phagocytes, they can internalize apoptotic neutrophils, apoptotic bodies, platelets and pathogens such as *Listeria monocytogenes* [30,31,32,33]. In our experiments, HMEC-1 did not internalize *Leishmania* promastigotes, and, to the best of our knowledge, there is no evidence of *Leishmania* phagocytosis by endothelial cells in vivo.

The involvement of the innate immune receptor TLR4 was investigated by using an anti-TLR4 antibody. TLR4, the sensing receptor for LPS, is expressed in immune and non-immune cells, including endothelial cells [34], being involved in the activation of the proinflammatory response and the production of cytokines. In the murine model of leishmaniasis, TLR4 is required for efficient parasite control [35] and in vitro studies demonstrated that GP29, a *L. donovani* derived glycoprotein, induced TNF-α and IL-12 production through TLR4 activation [36]. By showing a partial reduction of CXCL8 production, we can hypothesize that TLR4 is involved in CXCL8 production induced by *Leishmania*. The NF-κB pathway can be activated by pattern-recognition receptors, such as TLR, leading to the modulation of a large array of genes involved in inflammatory responses [37]. Artemisinin, an antimalarial agent, known to inhibit nuclear translocation of NF-kB complex [38] inhibited the production of CXCL-8 induced by *Leishmania*, suggesting the involvement of NF-κB pathway. However, since the reduction of CXCL-8 production was only partial, we cannot exclude that other signals or other TLRs could contribute to cell activation. It is known that *Leishmania* lipophosphoglycan (LPG), the major parasite ligand for macrophage adhesion, activates innate immune signaling pathways via TLR2 [39]. Furthermore, endosomal TLR9 can recognizes unmethylated CpG DNA sequences of *Leishmania* [40].

Interestingly, all three of the species used in this study, *L. infantum*, *L. tropica* and *L. brazilensis*, induced CXCL8 production. This indicates that the ability to stimulate endothelial cells is not species-specific, and not even related to the different pathogenesis, but it is a general feature of different *Leishmania* species. This contrasts with the host’s specific immunity to *Leishmania*, which is often species-specific and can either promote or control the infection [41]. However, being obligate intracellular parasites, promastigotes are rapidly destroyed in the extracellular tissues [42]. Therefore, all *Leishmania* species need to rapidly colonize host cells in order to survive and establish infection.

*L. infantum* promastigotes failed to induce CCL5 by HMEC-1 cells, but inhibited its production induced by TNF-α. This seems to be specific for CCL5, since CXCL8 production induced by TNF-α was not affected by *L. infantum*. CCL5, also known as RANTES, is important for the recruitment and development of Th1 cells, which are responsible for the control of the infection [43]. In addition, it attracts and activates many different immune cells including T cells, dendritic cells and NK cells to the sites of infection. Indeed, CCL5 induces IL-12 [44] and IFN-γ [45]. Moreover, it has been described that CCL5 contributes to the resistance to *L. major* infection [46]. In fact, in vivo treatment with Met-RANTES, an antagonist of CCR1 and CCR5, resulted in animals being more susceptible to the infection and in an increase in lesion size [46]. Therefore, the observed inhibition of CCL5 by the parasite, may strongly contribute to the establishment of the infection.

The presence of functional chemokines in the conditioned medium from endothelial cells treated with *Leishmania* promastigotes was confirmed with the transwell chemotaxis assay. The *Leishmania*-HMEC-1 conditioned medium recruited higher numbers of leukocytes than medium from unstimulated HMEC-1 cells. Furthermore, the total number of migrating leukocytes was comparable to that of leukocytes recruited by conditioned medium from HMEC-1 stimulated with LPS or TNF-α, two potent proinflammatory stimuli. After 2 h of chemotaxis, the migrated cells were mostly neutrophils in all studied groups. Neutrophils, the most abundant leukocyte subpopulation in the blood, express different surface receptors, which help them control their migration and behavior [47], and often serves as the first responders to a variety of inflammatory stimuli. After 24 h of migration, a higher percentage of monocytes was detected in conditioned media from unstimulated cells, whereas neutrophils still predominated in conditioned medium from *Leishmania*-stimulated cells. Macrophages represent the ultimate host cells for *Leishmania*, where parasites can survive and multiply.

In conclusion, our data provide novel insights into how *Leishmania* spp. promastigotes interact with endothelial cells, and generate a microenvironment able to attract phagocytic cells through the production of chemokines. Parasites must quickly invade phagocytic cells before losing their ability to infect. It is likely that a combination of signals from vector, parasite and host contribute to the early steps of natural infection. In addition to the sandfly saliva stimuli, the high production of CXCL8 by endothelial cells at the site of parasite deposition, may indeed contribute to the recruitment of PMNs, which provide shelter to parasites and allow them to survive and multiply, supporting the development of the disease. The concomitant reduction of CCL5 may further contribute to parasite survival and adaptation to the new host.

## 4. Materials and Methods

### 4.1. Leishmania spp. Culture

Promastigote stage of *L. infantum* (MHOM/TN/80/IPT1), *L. tropica* (MHOM/SY/2012/ISS3130) and *L. braziliensis* (MHOM/PE/2006/ISS2848) were cultured in Schneider’s Drosophila Medium (Lonza) supplemented with 10% heat-inactivated fetal calf serum (HyClone), 20 mM Hepes, and 2 mM *L*-glutamine at 23 °C.

### 4.2. Endothelial Cells Culture

The long-term cell line of dermal microvascular endothelial cells (HMEC-1) immortalized by SV 40 large T antigen [48] was kindly provided by the Center for Disease Control, Atlanta, GA. Cells were maintained in MCDB 131 medium supplemented with 10% fetal calf serum, 10 ng/mL of epidermal growth factor, 1 µg/mL of hydrocortisone, 2 mM glutamine, 100 units/mL of penicillin, 100 µg/mL of streptomycin and 20 mM Hepes buffer, pH 7.4.

### 4.3. Morphological Studies of Leishmania Promastigotes

Promastigotes of *L. infantum* were counted and 10^6^ parasites/well were seeded in 24-well flat bottom tissue culture clusters. Promastigotes were then cultured in standard conditions (complete Schneider’s Drosophila Medium, 23 °C) or in cell culture conditions (cell medium, 37 °C, 5% CO_2_) for 4 and 24 h. After incubation, parasites were recovered and a thin layer of parasites smeared on a slide. Slides were fixed with methanol and stained with Giemsa. Morphology was evaluated by light microscopy using a 100× oil immersion objective.

### 4.4. Cell and Leishmania spp. Viability

Cell and *Leishmania* spp. viability was evaluated using the 3-(4,5-dimethylthiazol-2-yl)-2,5-diphenyltetrazolium bromide (MTT) assay [49]. After incubation of the cells in different conditions, 20 μL of MTT solution (5 mg/mL in PBS) were added to the cells for 3 h at 37 °C in the dark. The supernatants were then discarded and the dark blue formazan crystals dissolved using 100 μL of lysis buffer containing 20% (wt/vol) sodium dodecylsulfate, 40% *N*,*N*-dimethylformamide (pH 4.7 in 80% acetic acid). The plates were then read on a Synergy 4 (Biotek) microplate reader at a test wavelength of 550 nm and at a reference wavelength of 650 nm.

### 4.5. Phagocytosis of Leishmania Promastigotes by Macrophages

THP-1 cells (human acute monocytic leukemia cell line) were maintained in RPMI 1640 supplemented with 10% FBS (EuroClone), 50 μM 2-mercaptoethanol, 20 mM Hepes, 2 mM glutamine, at 37 °C in 5% CO_2_. For *Leishmania* infections, THP-1 cells were plated at 5 × 10^4^ cells/well in 16-chamber Lab-Tek culture slides (Nunc) and treated with 0.1 μM phorbol myristate acetate (PMA, Sigma) for 48 h to achieve differentiation into macrophages. Cells were washed and infected with *Leishmania* spp. promastigotes at a macrophage:promastigote ratio of 1:10 for 24 h. The ability of the parasites to infect macrophages was examined after 4 h of promastigotes incubation in: (i) standard conditions (complete Schneider’s Drosophila Medium, 23 °C), (ii) cell culture conditions (cell medium, 37 °C, 5% CO_2_), (iii) co-incubation with HMEC-1 (1:10, cell:promastigote ratio) in cell culture conditions. After incubation, slides were fixed with methanol and stained with Giemsa. The percentage of infected macrophages was determined by light microscopy.

### 4.6. Endothelial Cells Treatment

HMEC-1 were seeded at 10^5^ cells/well in 24-well flat bottom tissue culture clusters. After overnight incubation, monolayers were exposed to stationary-phase promastigotes of *L. infantum* or *L. tropica* or *L. braziliensis* at different cells: *Leishmania* ratios (1:10, 1:5, 1:2.5) in a humidified CO_2_/air-incubator at 37 °C for 2 or 24 h. In some experiments, cells were stimulated with LPS (100 ng/mL) or TNF-α (100 U/mL). In other experiments, neutralizing anti-TLR4 IgG monoclonal antibodies (InvivoGen) at 0.5 µM were added to the cells 30 min before the addition of *Leishmania* promastigotes (1:10 ratio). LPS (100 ng/mL) was used as control ligand of TLR-4. In some experiments, HMEC-1 were treated with *Leishmania* promastigotes (1:10 ratio) in the presence of artemisinin (10 µM).

At the end of each treatment, supernatants were collected and used for chemokine determinations or transwell migration assay (conditioned media).

### 4.7. Chemokine Determination

CXCL8 and CCL5 were measured in cell supernatants by DuoSet ELISA Kit (R&D System) following the manufacturer’s instructions.

### 4.8. Isolation of Peripheral Blood Lymphocytes (PBL) and Transwell Migration Assay

PBLs were isolated from fresh peripheral blood of human donors. Blood was diluted 1:1 with RPMI1640, layered on cold Ficoll-Paque and centrifuged at 500× *g* for 30 min [50]. PBLs were recovered, washed with RPMI and counted.

Supernatants from endothelial cells treated with different stimuli (conditioned media) were introduced into the lower compartment of a 24 transwell plate (3 μm pores). PBLs (1 × 10^5^) were added to the upper compartment and the transwell plate was incubated for 2 and 24 h at 37 °C in 5% CO_2_ incubator. At the end of incubation, the insert was carefully taken out to remove non-migrated cells. Migrated cells on the lower side were counted by trypan blue using light microscopy and 2 × 10^5^ cells in 100 μL were used for cytospin preparation. Cells were centrifuged at 500 rpm for 5 min in a cytospin centrifuge. Slides were fixed with methanol, stained with Giemsa and the percentages of the different leucocyte subpopulations counted by microscopic observation.

### 4.9. Statistical Analyses

All data were obtained from three independent experiments and the results are shown as mean ± standard deviation or as a representative experiment. Differences between groups were analyzed for statistical significance by using 1-way or 2-way ANOVA tests followed by post hoc multiple comparison tests (Dunnett’s, Sidak’s or Tukey’s). Statistical significance was set at *p* < 0.05.

## Data Availability

The data presented in this study are available on request from the corresponding author.

## References

[1] World Health Organization Fact-Sheet Leishmaniasis. https://www.who.int/news-room/fact-sheets/detail/leishmaniasis.

[2] Burza S., Croft S.L., Boelaert M. (2018). Leishmaniasis. Lancet.

[3] Peters N.C., Egen J.G., Secundino N., Debrabant A., Kimblin N., Kamhawi S., Lawyer P., Fay M.P., Germain R.N., Sacks D. (2008). In vivo imaging reveals an essential role for neutrophils in leishmaniasis transmitted by sand flies. Science.

[4] Peters N.C., Sacks D.L. (2009). The impact of vector-mediated neutrophil recruitment on cutaneous leishmaniasis. Cell Microbiol..

[5] Ribeiro-Gomes F.L., Peters N.C., Debrabant A., Sacks D.L. (2012). Efficient capture of infected neutrophils by dendritic cells in the skin inhibits the early anti-leishmania response. PLoS Pathog..

[6] Thalhofer C.J., Chen Y., Sudan B., Love-Homan L., Wilson M.E. (2011). Leukocytes infiltrate the skin and draining lymph nodes in response to the protozoan Leishmania infantum chagasi. Infect. Immun..

[7] Wilson M.E., Innes D.J., Sousa A.D., Pearson R.D. (1987). Early histopathology of experimental infection with Leishmania donovani in hamsters. J. Parasitol..

[8] Pompeu M.L., Freitas L.A., Santos M.L., Khouri M., Barral-Netto M. (1991). Granulocytes in the inflammatory process of BALB/c mice infected by Leishmania amazonensis. A quantitative approach. Acta Trop..

[9] Beil W.J., Meinardus-Hager G., Neugebauer D.C., Sorg C. (1992). Differences in the onset of the inflammatory response to cutaneous leishmaniasis in resistant and susceptible mice. J. Leukoc. Biol..

[10] Müller K., van Zandbergen G., Hansen B., Laufs H., Jahnke N., Solbach W., Laskay T. (2001). Chemokines, natural killer cells and granulocytes in the early course of Leishmania major infection in mice. Med. Microbiol. Immunol..

[11] Guimaraes-Costa A.B., Shannon J.P., Waclawiak I., Oliveira J., Meneses C., de Castro W., Wen X., Brzostowski J., Serafim T.D., Andersen J.F. (2021). A sand fly salivary protein acts as a neutrophil chemoattractant. Nat. Commun..

[12] Ribeiro-Gomes F.L., Sacks D. (2012). The influence of early neutrophil-Leishmania interactions on the host immune response to infection. Front. Cell. Infect. Microbiol..

[13] Dey R., Joshi A.B., Oliveira F., Pereira L., Guimarães-Costa A.B., Serafim T.D., de Castro W., Coutinho-Abreu I.V., Bhattacharya P., Townsend S. (2018). Gut Microbes Egested during Bites of Infected Sand Flies Augment Severity of Leishmaniasis via Inflammasome-Derived IL-1β. Cell Host Microbe.

[14] Nauseef W.M., Borregaard N. (2014). Neutrophils at work. Nat. Immunol..

[15] Regli I.B., Passelli K., Hurrell B.P., Tacchini-Cottier F. (2017). Survival Mechanisms Used by Some Leishmania Species to Escape Neutrophil Killing. Front. Immunol..

[16] Aga E., Katschinski D.M., van Zandbergen G., Laufs H., Hansen B., Müller K., Solbach W., Laskay T. (2002). Inhibition of the Spontaneous Apoptosis of Neutrophil Granulocytes by the Intracellular Parasite *Leishmania major*. J. Immunol..

[17] van Zandbergen G., Klinger M., Mueller A., Dannenberg S., Gebert A., Solbach W., Laskay T. (2004). Cutting edge: Neutrophil granulocyte serves as a vector for Leishmania entry into macrophages. J. Immunol..

[18] Viola A., Luster A.D. (2008). Chemokines and their receptors: Drug targets in immunity and inflammation. Annu. Rev. Pharm. Toxicol..

[19] Baggiolini M. (2001). Chemokines in pathology and medicine. J. Intern. Med..

[20] Abdeladhim M., Kamhawi S., Valenzuela J.G. (2014). What’s behind a sand fly bite? The profound effect of sand fly saliva on host hemostasis, inflammation and immunity. Infect. Genet. Evol..

[21] Alcolea P.J., Alonso A., Gómez M.J., Sánchez-Gorostiaga A., Moreno-Paz M., González-Pastor E., Toraño A., Parro V., Larraga V. (2010). Temperature increase prevails over acidification in gene expression modulation of amastigote differentiation in Leishmania infantum. BMC Genom..

[22] Zilberstein D., Shapira M. (1994). The role of pH and temperature in the development of Leishmania parasites. Annu. Rev. Microbiol..

[23] Mittra B., Cortez M., Haydock A., Ramasamy G., Myler P.J., Andrews N.W. (2013). Iron uptake controls the generation of Leishmania infective forms through regulation of ROS levels. J. Exp. Med..

[24] Alzate J.F., Arias A.A., Moreno-Mateos D., Alvarez-Barrientos A., Jiménez-Ruiz A. (2007). Mitochondrial superoxide mediates heat-induced apoptotic-like death in Leishmania infantum. Mol. Biochem. Parasitol..

[25] Puentes F., Diaz D., Hoya R.D., Gutíerrez J.A., Lozano J.M., Patarroyo M.E., Moreno A. (2000). Cultivation and characterization of stable Leishmania guyanensis complex axenic amastigotes derived from infected U937 cells. Am. J. Trop. Med. Hyg..

[26] van Zandbergen G., Hermann N., Laufs H., Solbach W., Laskay T. (2002). Leishmania promastigotes release a granulocyte chemotactic factor and induce interleukin-8 release but inhibit gamma interferon-inducible protein 10 production by neutrophil granulocytes. Infect. Immun..

[27] Kumar R., Bumb R.A., Salotra P. (2010). Evaluation of localized and systemic immune responses in cutaneous leishmaniasis caused by Leishmania tropica: Interleukin-8, monocyte chemotactic protein-1 and nitric oxide are major regulatory factors. Immunology.

[28] Costa D.J., Favali C., Clarêncio J., Afonso L., Conceição V., Miranda J.C., Titus R.G., Valenzuela J., Barral-Netto M., Barral A. (2004). Lutzomyia longipalpis salivary gland homogenate impairs cytokine production and costimulatory molecule expression on human monocytes and dendritic cells. Infect. Immun..

[29] Utgaard J.O., Jahnsen F.L., Bakka A., Brandtzaeg P., Haraldsen G. (1998). Rapid secretion of prestored interleukin 8 from Weibel-Palade bodies of microvascular endothelial cells. J. Exp. Med..

[30] Gao C., Xie R., Li W., Zhou J., Liu S., Cao F., Liu Y., Ma R., Si Y., Bi Y. (2013). Endothelial cell phagocytosis of senescent neutrophils decreases procoagulant activity. Thromb. Haemost..

[31] Dini L., Lentini A., Diez G.D., Rocha M., Falasca L., Serafino L., Vidal-Vanaclocha F. (1995). Phagocytosis of apoptotic bodies by liver endothelial cells. J. Cell Sci..

[32] Ma R., Xie R., Yu C., Si Y., Wu X., Zhao L., Yao Z., Fang S., Chen H., Novakovic V. (2017). Phosphatidylserine-mediated platelet clearance by endothelium decreases platelet aggregates and procoagulant activity in sepsis. Sci. Rep..

[33] Rengarajan M., Hayer A., Theriot J.A. (2016). Endothelial Cells Use a Formin-Dependent Phagocytosis-Like Process to Internalize the Bacterium Listeria monocytogenes. PLoS Pathog..

[34] Taylor K.R., Trowbridge J.M., Rudisill J.A., Termeer C.C., Simon J.C., Gallo R.L. (2004). Hyaluronan fragments stimulate endothelial recognition of injury through TLR4. J. Biol. Chem..

[35] Kropf P., Freudenberg N., Kalis C., Modolell M., Herath S., Galanos C., Freudenberg M., Müller I. (2004). Infection of C57BL/10ScCr and C57BL/10ScNCr mice with Leishmania major reveals a role for Toll-like receptor 4 in the control of parasite replication. J. Leukoc. Biol..

[36] Paul J., Naskar K., Chowdhury S., Alam M.N., Chakraborti T., De T. (2014). TLR4-mediated activation of MyD88 signaling induces protective immune response and IL-10 down-regulation in Leishmania donovani infection. Indian J. Biochem. Biophys..

[37] Liu T., Zhang L., Joo D., Sun S.C. (2017). NF-κB signaling in inflammation. Signal Transduct. Target. Ther..

[38] Aldieri E., Atragene D., Bergandi L., Riganti C., Costamagna C., Bosia A., Ghigo D. (2003). Artemisinin inhibits inducible nitric oxide synthase and nuclear factor NF-kB activation. FEBS Lett..

[39] Sacramento L.A., da Costa J.L., de Lima M.H., Sampaio P.A., Almeida R.P., Cunha F.Q., Silva J.S., Carregaro V. (2017). Toll-Like Receptor 2 Is Required for Inflammatory Process Development during. Front. Microbiol..

[40] Gupta C.L., Akhtar S., Waye A., Pandey N.R., Pathak N., Bajpai P. (2015). Cross talk between Leishmania donovani CpG DNA and Toll-like receptor 9: An immunoinformatics approach. Biochem. Biophys. Res. Commun..

[41] Rossi M., Fasel N. (2018). How to master the host immune system? Leishmania parasites have the solutions!. Int. Immunol..

[42] Sacks D.L., Perkins P.V. (1984). Identification of an infective stage of Leishmania promastigotes. Science.

[43] Weber C., Weber K.S.C., Klier C., Gu S., Wank R., Horuk R., Nelson P.J. (2001). Specialized roles of the chemokine receptors CCR1 and CCR5 in the recruitment of monocytes and TH1-like/CD45RO+T cells. Blood.

[44] Aliberti J., Reis e Sousa C., Schito M., Hieny S., Wells T., Huffnagle G.B., Sher A. (2000). CCR5 provides a signal for microbial induced production of IL-12 by CD8α+ dendritic cells. Nat. Immunol..

[45] Makino Y., Cook D.N., Smithies O., Hwang O.Y., Neilson E.G., Turka L.A., Sato H., Wells A.D., Danoff T.M. (2002). Impaired T cell function in RANTES-deficient mice. Clin. Immunol..

[46] Santiago H.C., Oliveira C.F., Santiago L., Ferraz F.O., de Souza D.G., de-Freitas L.A., Afonso L.C., Teixeira M.M., Gazzinelli R.T., Vieira L.Q. (2004). Involvement of the chemokine RANTES (CCL5) in resistance to experimental infection with Leishmania major. Infect. Immun..

[47] Futosi K., Fodor S., Mócsai A. (2013). Neutrophil cell surface receptors and their intracellular signal transduction pathways. Int. Immunopharmacol..

[48] Ades E.W., Candal F.J., Swerlick R.A., George V.G., Summers S., Bosse D.C., Lawley T.J. (1992). HMEC-1: Establishment of an immortalized human microvascular endothelial cell line. J. Investig. Derm..

[49] Baiocco P., Ilari A., Ceci P., Orsini S., Gramiccia M., Di Muccio T., Colotti G. (2011). Inhibitory Effect of Silver Nanoparticles on Trypanothione Reductase Activity and Leishmania infantum Proliferation. ACS Med. Chem. Lett..

[50] Kuhns D.B., Priel D.A.L., Chu J., Zarember K.A. (2015). Isolation and Functional Analysis of Human Neutrophils. Curr. Protoc. Immunol..

